# Co-designing embodied extended reality telehealth for veteran mental health: from movement care to whole health

**DOI:** 10.3389/fdgth.2026.1952753

**Published:** 2026-10-01

**Authors:** Aviv Elor, Maxim Kuznetsov, Laura Kusumoto, Jared Duval, Kedron Burnett, Felicia Skelton, Hilary Touchett

**Affiliations:** 1 Immergo Labs, Inc., Mountain View, CA, United States; 2 School of Informatics, Computing, and Cyber Systems, Northern Arizona University, Flagstaff, AZ, United States; 3 VA Clinical Resource Hub National Telehealth Program, US Department of Veterans Affairs, Boise, ID, United States; 4 Spinal Cord Injury Care Line, Michael E. DeBakey VA Medical Center, Houston, TX, United States; 5 Ben Taub Department of Physical Medicine and Rehabilitation, Baylor College of Medicine, Houston, TX, United States; 6 Houston VA HSR&D Center for Innovations in Quality, Effectiveness and Safety (IQuESt), Michael E. DeBakey VA Medical Center, Houston, TX, United States; 7 Department of Medicine, Baylor College of Medicine, Houston, TX, United States; 8 South Central Mental Illness Research, Education, Clinical Center (a Virtual Center), Houston, TX, United States

**Keywords:** codesign, extended reality, mental health, participatory design, telehealth, veterans, virtual reality, whole health

## Abstract

**Introduction:**

Veterans in rural and underserved communities face persistent care barriers from provider shortages, travel burdens, and the digital divide. Within the Department of Veterans Affairs (VA), the Clinical Resource Hub (CRH) National Telehealth Program delivers primary, mental health, specialty, surgical, and rehabilitative care into veterans’ homes and rural clinics, while VA Immersive scales extended reality (XR) across the system. Embodied XR telehealth, where provider and patient meet as full-body avatars in a shared immersive environment, intersects these programs and introduces new design requirements. This study extends our embodied XR codesign series from physical rehabilitation to mental health and whole health.

**Methods:**

Stakeholders from VA, academia, and industry (N=30), drawn from Boise and other participating CRHs, joined a 3-day codesign at the Boise VA Medical Center in June 2026, including shadowing telehealth workflows, a full-day workshop with five teams, and a half-day debrief with program leads. Seven usability task ratings, 164 four-axis ideas (Raise, Reduce, Eliminate, Create), 95 debrief proposals, 188 prioritization votes, and 19 discussion transcripts were analyzed through descriptive statistics and reflexive thematic analysis.

**Results:**

Perceived task ease was high among a cohort with mixed VR experience across seven tasks (tutorial, joining a telehealth room, exercise completion, movement recording, environment and interactable customization, and spatial drawing), each above 4.1 of 5 on the Single Ease Question, led by customizing virtual environments (M=4.84±0.37). Stakeholders most endorsed minimalist defaults, onboarding resources, and out-of-the-box readiness, followed by embodiment with personalized environments, disability representation, and veteran identity markers. The debrief prioritized breathing and grounding, graded exposure, psychoeducation, and whole-health movement, delivered through headsets paired to an in-clinic tablet or monitored home deployment. Infrastructure compatibility, workforce fit, and governance emerged as pervasive constraints. Workshop satisfaction was high (M=4.82±0.53 of 5) with 88% requesting follow-up; platform-specific ratings were favorable but lower and more variable (e.g., user-friendliness M=3.76±0.97 of 5).

**Discussion:**

We propose candidate design principles for extending an embodied XR telehealth platform, built for physical rehabilitation, to mental health and whole health care for veterans, and discuss implications for platform design, piloting, and deployment.

## 1 Introduction

The benefits of digital health innovation are not evenly distributed (1–4). Access to telehealth, remote monitoring, and immersive technologies has grown in potential, but older adults, rural communities, people with disabilities, and low-income groups remain disproportionately limited in connectivity, device access, and digital literacy. Innovations that do not address these limitations may reinforce, rather than reduce, existing health disparities (5, 6). In the United States, veterans are frequently overrepresented among these populations and can experience greater health burdens than the general population, attributable to service-connected disabilities, chronic pain, and mental health needs (7, 8).

The Veterans Health Administration, the largest integrated healthcare system in the United States, serves over 9 million enrolled veterans at more than 1,000 care sites and has developed extensive infrastructure to enhance access to healthcare innovations (9). One such initiative is the Clinical Resource Hub (CRH) program, a national contingency-staffing and telehealth network that delivers care across multiple service lines, including primary care, mental health, specialty medicine, surgical care, and rehabilitation, through regional hubs to veterans’ homes and rural clinics, with a specific focus on rural and under-resourced sites (10–12). The present study focuses specifically on CRH’s primary care and mental health service lines, the two most directly engaged by our participating stakeholders. Another is Department of Veterans Affairs (VA) Immersive, which has deployed extended reality (XR), the umbrella term spanning virtual, augmented, and mixed reality,^1^ across more than 170 facilities in one of the world’s largest clinical deployments to date, most commonly used for pain management, physical rehabilitation, and mental healthcare (9). These two distribution infrastructures have developed concurrently. CRH enables clinicians to provide care remotely via telephone and video visits, while VA Immersive facilitates the delivery of immersive interventions in clinical settings and, increasingly, in veterans’ homes.

Immersive virtual reality (VR) has an established evidence base in mental health, including exposure therapy for anxiety and posttraumatic stress disorder (PTSD), guided breathing and relaxation, and behavioral activation (13–17). Telehealth-based VR clinics have begun to demonstrate that these interventions can be delivered remotely (18). An embodied XR telehealth platform introduces a representational component to the clinical encounter by enabling clinicians and veterans to interact within a shared virtual environment as full-body avatars. It combines the advantages of telehealth and immersive technologies. Traditional 2D video telehealth can support effective interpersonal care with outcomes comparable to in-person delivery (19, 20), yet it often remains limited for embodied, movement-based care, constraining non-verbal signals, spatial guidance, and objective movement assessment (21, 22). With 3D representation and avatar embodiment, XR can afford copresence and spatial interaction (23, 24), which may further strengthen engagement and collaborative interaction while restoring the embodied channels that movement and whole healthcare require. However, there is limited experience deploying this type of platform in the VA, creating an opportunity to study the design requirements.

The requirements are further constrained by the need for movement-based care to work within an integrated telehealth network, on government-managed deployments, and over veterans' home connectivity. Additionally, the platform’s use must align with the workload capacities of the national provider workforce. Providers report interest in VR-based telehealth, but they often have little direct experience (25). Implementation research consistently demonstrates that technology-centric design is unsuccessful when it fails to consider the adopting system (26).

### 1.1 Context and motivation

This study expands upon a codesign lineage that originated in physical rehabilitation to include mental health and whole health. Our first stakeholder workshop engaged private-practice clinicians and patients (N=24) to develop six design principles for embodied XR telerehabilitation, including embodied guidance with real-time feedback, progressive complexity with minimalist defaults, and adaptive accessibility through multimodal input (27). A subsequent, smaller workshop involving VA rehabilitation clinicians and design researchers (N=8; four providers, four design researchers) at a Houston VA Medical Center (VAMC) revealed that integrated-network stakeholders may prioritize accessibility and adaptation over the interface simplicity emphasized in private practice. Embodied XR telehealth platforms should be configurable based on the context and patient population needs (28). Both studies build upon earlier findings that 2D telehealth often leaves providers feeling more like “life coaches” than clinicians, due to a lack of embodied interaction and objective measurement (21).

Building on these findings, extending an embodied XR telerehabilitation platform to support mental healthcare is motivated on three grounds. First, movement is inherently therapeutic for mental health. Physical activity is positively associated with improved mental health outcomes and has been shown to benefit individuals experiencing depression and anxiety (29, 30). A movement care platform can therefore facilitate holistic mental health treatment by integrating mind-and-body-connected movement with clinician-delivered interventions. Second, the VA’s Whole Health initiative reorients care toward holistic, personalized approaches that focus on what matters to the veteran, rather than solely on their medical conditions. This initiative provides the institutional framework for such comprehensive care (31, 32). Third, as a delivery system whose mandate is to reach underserved veterans, CRH is purpose-built to provide remote care in veterans’ homes, which affects clinician workflows. Design requirements must be coproduced with this workforce that delivers care specifically for CRH (33, 34).

Accordingly, the present study extends the codesign series in multiple ways: from physical rehabilitation to mental health and whole health; from clinic-based providers to a national telehealth network with the veteran’s home as the default care setting; and from single-day or half-day formats to a 3-day structure incorporating shadowing, codesign, and deployment planning. Together, these extensions yield broader insights into embodied XR telehealth platforms that must serve multiple care use cases within an integrated network.

### 1.2 Study approach and objectives

We conducted a 3-day codesign at the Veterans Integrated Service Network (VISN) 20 CRH on the Boise VAMC campus in June 2026, convened by Immergo Labs, in collaboration with the VA CRH National Telehealth Program and the VA Houston Center for Innovations in Quality, Effectiveness and Safety (IQuESt) under a quality improvement (QI) designation. The workshop addressed the following three primary study questions:

1. *SQ1 (Current state):* What access, infrastructure, workforce, and safety conditions already shape telehealth mental healthcare in an integrated system, before any headset is introduced?
2. *SQ2 (Design priorities):* When CRH stakeholders ideate and prioritize an embodied XR telehealth platform for mental and whole health, what do they articulate, propose, vote for, and rank, and where do these signals diverge?
3. *SQ3 (Build and deployment paths):* What capabilities do stakeholders identify as the highest priorities for initial development, and what institutional pathways do they recommend for piloting?

### 1.3 Contributions of this study

This study was conducted as a QI activity within a national VA telehealth program. It reports formative design insights from one cohort of 30 stakeholders, comprising clinicians, academics, and industry participants. No veterans participated directly, so all findings are framed throughout as stakeholder perceptions of veteran needs rather than as veteran-reported requirements. Specifically, this study contributes the following:

1. *Empirical:* Codesign findings from 30 stakeholders affiliated with a national VA telehealth program, organized by day and categorized by distinct evidence signals (what participants said, proposed, voted for, and prioritized) for this cohort.
2. *Theory:* Candidate design principles for embodied XR telehealth in mental health and whole health integrated care, derived from this cohort’s input and read alongside those established in previous physical rehabilitation codesigns.
3. *Methodology:* A 3-day codesign methodology that integrates shadowing, Single Ease Question (SEQ) usability tasks, four-axis framework analysis, cross-team prioritization voting, and action mapping, with agenda tables provided for adaptation by other research teams.

## 2 Related work

### 2.1 Embodied presence beyond 2D video

Telepresence is a function of vividness and interactivity rather than any specific hardware (23). Immersive XR can extend videoconferencing along both dimensions by giving each party a tracked, full-body avatar in a shared spatial environment. The sense of embodiment decomposes into body ownership, agency, and self-location (24), and higher embodiment intensifies the emotional response to virtual stimuli (35), a mechanism directly relevant to mental health intervention, in which emotional engagement is often a key mechanism of change (36). Immersive learning research further suggests that presence and agency mediate outcomes, while cognitive load moderates them (37, 38), thereby framing onboarding and interface simplicity as clinical variables rather than conveniences.

### 2.2 Virtual reality for mental and whole health

VR has moved from promise to evidence in mental health assessment and treatment (13, 17). Meta-analytic work supports VR exposure therapy for anxiety and related disorders (15), with randomized trials in veteran and active-duty PTSD populations showing efficacy comparable to imaginal exposure (39, 40). VR breathing and relaxation interventions show measurable mental health benefits (16, 41), and internet-delivered behavioral activation is effective for depression (42). Beyond symptom-targeted care, physical activity is itself an effective intervention for depression and anxiety (29, 30), which matters for a platform whose lineage is movement care: tai chi, yoga, and guided movement surfaced repeatedly in our workshop as whole health content. The VA’s Whole Health model provides the institutional frame for that breadth (31, 32). Early telehealth-based VR clinics demonstrate remote delivery at fidelity (18), while provider and client studies report interest tempered by inexperience and onboarding concern (25, 43).

### 2.3 Avatars, identity, and personalization in therapy

In XR healthcare, avatar design often serves more than a cosmetic role. Avatar-based therapy has been suggested to operationalize the clinical support of a personalized virtual representation, with trial evidence for reducing distress from auditory hallucinations (44, 45). Self-representation is also suggested to change behavior: the Proteus effect posits that people conform to the identities their avatars carry, which can influence their behavior (46), and embodied self-compassion paradigms use avatar perspective-taking therapeutically (47). This cuts both ways, since embodiment can also amplify harm, and disability scholarship cautions that immersive technology often erases or misrepresents minority bodies, so it is critical to design representation with informed intent (48). Veteran identity markers, disability representation, and culturally meaningful environments are design elements with clinical and equity implications.

### 2.4 Telemental health: effectiveness, friction, and warm handoffs

Videoconferenced mental healthcare can be as effective as in-person care (19), sustains working alliance (20), and scaled rapidly during the COVID-19 pandemic (49, 50). The VA has trial evidence that home-based telehealth delivery of trauma-focused therapy can be non-inferior to in-person delivery (51). Yet friction persists: clinicians report that 2D telehealth constrains non-verbal signals and assessment (21, 22), and premature discontinuation remains a structural weakness of psychotherapy generally (52). Warm handoffs, the live introduction of a patient to a behavioral health provider, can measurably improve initial engagement in integrated care (53–55). An embodied telehealth room is a strong potential candidate infrastructure for making the telehealth warm handoff feel more like an introduction than a transfer, a use case our codesign participants raised unprompted.

### 2.5 Integrated veteran care, CRH, and rural digital health equity

The CRH program was designed to buffer staffing gaps and extend specialty and mental healthcare to under-resourced, often rural VA sites through regional telehealth hubs (10, 11), including geriatric mental health reach into rural communities (12). Implementation studies of VA telemental health identify recurring barriers of technology provisioning, connectivity, workload, and clinician buy-in (8), and veterans with disabilities articulate distinct needs and ambivalences about telemental care (7). Digital health equity frameworks converge on the same determinants: access, digital literacy, trust, and design that survives poor connectivity (5, 6, 56). XR telehealth platforms for this population must address these determinants as primary requirements.

### 2.6 Codesign in digital health

Codesign engages end users as partners in shaping products and services (33), with healthcare traditions in experience-based codesign (57) and a growing evidence base on how codesign is conducted and reported in health (58–60). In healthcare XR specifically, stakeholder cocreation studies emphasize authentic engagement, balanced participant composition, and organizational rigor (34), and clinician-centered design has shaped therapeutic XR systems (61). Consistent with our prior workshops, we favor lightweight evaluation instruments, the Single Ease Question (SEQ) (62) and structured four-axis analysis adapted from strategy canvas methods (63), that preserve collaborative engagement over measurement burden (27).

### 2.7 A brief background on Immergo

Immergo Labs,^2^ a startup funded by the United States National Science Foundation (NSF) Small Business Innovation Research (SBIR) program, has developed an XR movement care platform informed by nearly a decade of academic XR rehabilitation research (21, 27, 61, 64). The platform, currently designed around physical rehabilitation, runs on consumer VR headsets and pairs two modes of care: live telehealth appointments in which a movement care provider and client meet as full-body tracked avatars in a shared virtual room, and asynchronous, gamified exercise programs that clients complete on their own with clinician-authored guidance and movement assessment. In VR, users can also customize environments and 3D content, record and replay movements, annotate in shared space, and complete assigned movement programs; a companion web application, also embedded inside the VR application, supports scheduling, avatar customization, camera-based full-body tracking, measure review, and program assignment. At the time of this codesign, the platform was deployed in private practice and under evaluation in a VA usability study with veteran participants for physical therapy. The present study is the third in the codesign series and the first to target mental health and whole health through a national telehealth program for one of the largest integrated healthcare networks in the United States, rather than for a single facility’s providers.

## 3 Materials and methods

### 3.1 Approach and rationale

This study employs codesign as its primary methodological lens, defined as a subset of participatory design that emphasizes direct collaboration between end users and designers to shape product features and interactions (33, 57). Consistent with our prior workshops (27), we combine lightweight quantitative instruments (SEQ post-task ratings (62)) with structured qualitative frameworks (four-axis analysis (34, 63), within-team importance ranking, and cross-team dot voting) to preserve collaborative engagement over measurement burden. A convergent mixed-methods evaluation combined these instruments with reflexive thematic analysis of session transcripts and workshop artifacts (65, 66). The three-phase structure deliberately sequences empathy building (Day 1 shadowing), divergent evaluation and ideation (Day 2 codesign workshop), and convergent synthesis with committed action (Day 3 debrief mapping), extending the single-day and half-day formats of our prior workshops to ground design activity in observed practice. The activity was determined to be a quality improvement VA operations activity by the Michael E. DeBakey VAMC (Houston) Research and Development Committee and did not require IRB review. Reporting is inspired by SQUIRE 2.0 guidance for quality improvement studies (67).

### 3.2 Setting and partners

The codesign took place from 23 to 25 June 2026, at the VISN 20 CRH on the Boise VAMC campus, convened by Immergo Labs with the VA CRH National Telehealth Program team and VA Houston VAMC IQuESt team. Boise is a VA CRH-served site within a substantially rural catchment, where participating providers deliver a high share of their care via telehealth to veterans’ homes, making it a fit-for-purpose setting for studying home-reaching XR telehealth. While VISN 20 hosted the event, participating CRH stakeholders traveled from several other regional CRH hubs, with representation spanning the National CRH Program Office and multiple VISNs, including VISNs 12, 16, 19, 20, and 21, rather than a single site’s staff alone. Activities used two conference rooms for workshop and debrief sessions and a simulation room housing CRH-deployed telehealth carts and peripherals for technology tours and shadowed sessions.

### 3.3 Participants

In total, 30 stakeholders participated in five mixed teams: 21 VA CRH stakeholders spanning primary care, primary care mental health integration, psychology and behavioral health, nursing, social work, pharmacy, medical support, and program leadership; 4 subject matter experts (SMEs) from academic and industry backgrounds spanning older adults, accessibility, user research, and XR; and 5 Immergo product team members serving as table coaches and a floating moderator. Each team included a table coach and three to five VA stakeholders, with SMEs distributed across teams (see Table 1 for composition). A floating Immergo moderator, with a PhD in human-computer interaction and over 8 years of user research experience in academic, industry, and government settings, coordinated timing and facilitation for all teams. As noted in Section 3.2, participating CRH stakeholders held nationally distributed CRH roles across multiple VISNs, including national program leadership. Veterans, as a protected population within the VA, were not directly involved due to the activity’s quality improvement designation. The workforce responsible for deploying new modalities served as the initial codesign population. Veteran-inclusive codesign will be conducted following required human-subjects approvals.

**Table 1:** Team composition across the 3-day codesign (N=30), with discipline tags per participant.

Team	Facilitator	VA stakeholders (discipline tags)	Embedded SME
Team 1	Product coach (product management)	3: Nursing; psychology/behavioral health; social work; medical support	Industry/older adult expert
Team 2	Product coach (design)	5: Physician/leadership (×2); pharmacy/leadership; psychology/leadership; leadership	None
Team 3	Product coach (product operations)	4: Physician/leadership; psychology/behavioral health; leadership (×2)	HCI/innovation strategist
Team 4	Product coach (HCI/engineering)	5: Nursing; medical support/leadership; social work; physician; medical support	None
Team 5	SME coach (academic HCI/XR user researcher)	4: Psychology/behavioral health; nursing; pharmacy; leadership	Academic HCI/XR accessibility

Each team paired one facilitator with its VA stakeholders; one Immergo product moderator (an HCI/user researcher with 8+ years of experience across academic, industry, and government settings) floated among all teams.

An anonymous preworkshop survey (n=18; 17 of 21 VA stakeholders and one SME) established a cohort baseline (see Table 2). Respondents were 25 to over 65 years of age and 83% were women, with 83% (15 of 18) reporting 10 or more years in their profession. The cohort was telehealth-saturated, delivering a mean of 81% (median 94%) of their care via telehealth, predominantly into the veteran’s home rather than a clinic room, and spanned the full range of VR experience, from never (28%) and limited (22%) through moderate (6%) to experienced (22%) and highly experienced (22%). The methodology was new to many: 78% had never participated in a codesign activity before. This profile of deep telehealth experience paired with mixed VR experience, with half of respondents at never-to-limited exposure, matches similar previous studies for telemental health providers (25).

**Table 2:** Preworkshop cohort baseline (N=18, 17 out of 21 VA stakeholders and one SME).

Characteristic	Value
Age	25–34: 6%; 35–44: 28%; 45–54: 39%; 55–64: 22%; 65 or older: 6%
Sex	Female 83%; male 17%
Years in profession	10+ years: 83% (15 of 18)
Disciplines (multiselect)	Social work 22%; specialty physician 17%; nursing 17%; plus psychology, advanced practice, primary care, pharmacy, rehabilitation, and program leadership
Care delivered via telehealth	Mean 81%, median 94% of care; modality mix: video into non-VA locations (home) 63%, in-person 18%, telephone 10%, clinic-based video telehealth 8%
Technology comfort (0–5)	M=4.2±1.29
Warm-handoff comfort (1–5)	M=4.2±1.15
VR experience	Never 28%; limited 22%; moderate 6%; experienced 22%; highly experienced 22%
Prior codesign experience	No 78%; yes 22%

Technology comfort and warm-handoff comfort were single-item self-ratings of comfort adopting new technology in clinical workflow (0–5) and comfort conducting warm handoffs over telehealth (1–5), with higher values indicating greater comfort.

### 3.4 Multiday codesign structure

Each of the 3 days used a different session type (Figure 1). Day 1 focused on current practice through shadowing. Day 2 was the generative codesign workshop. Day 3 focused on synthesis and action. Table 3 provides the full agenda for adaptation by other research teams. All table coaches were briefed before the workshop and had prior experience facilitating codesign sessions (27). Facilitation followed the shared agenda and the printed worksheets in the Appendix as standardized prompts; coaches were instructed to elicit and record participant ideas rather than propose or evaluate them, with embedded SMEs and VA investigators observing across teams.

**Figure 1:**
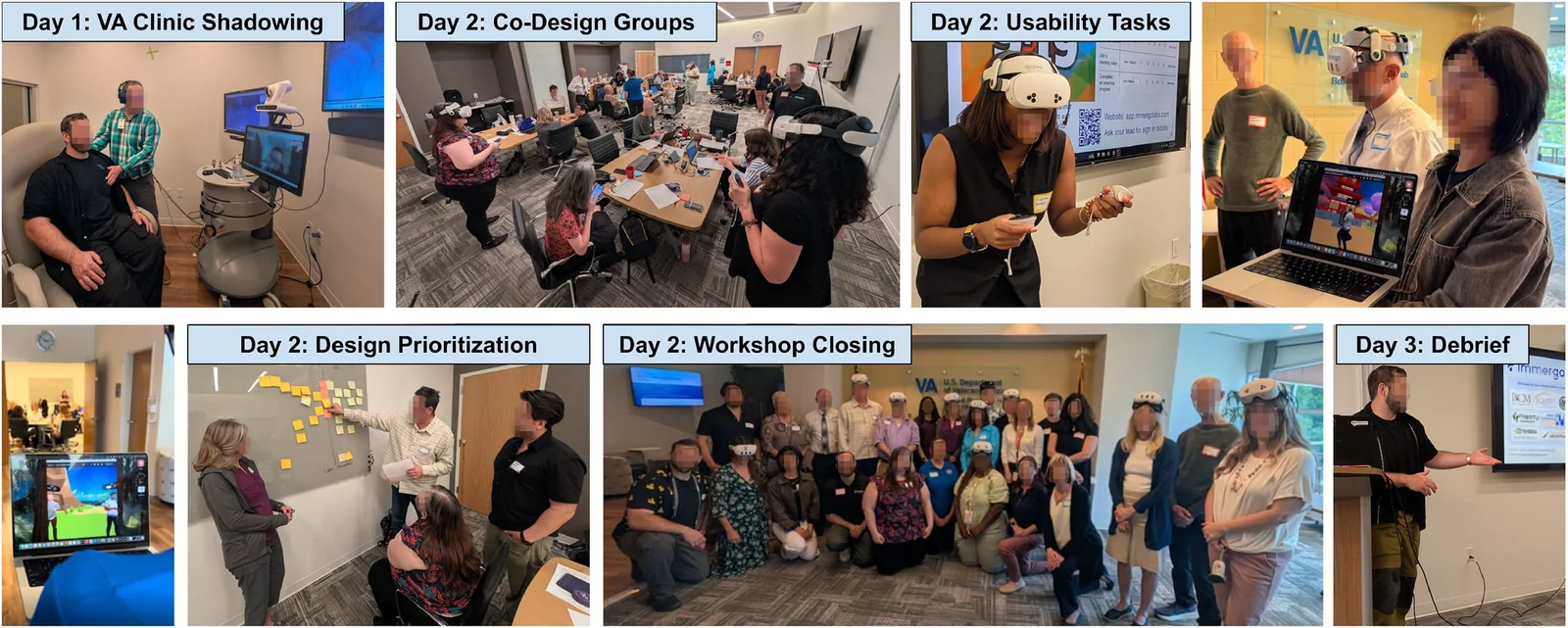
Overview of the 3-day codesign at the VISN 20 CRH Boise VAMC campus: Day 1 shadowing of current VA CRH telehealth workflows, Day 2 full-day codesign workshop with five mixed teams (usability tasks, four-axis ideation, and prioritization voting), and Day 3 debrief with team readouts and action mapping. The full agenda is given in Table 3.

**Table 3:** Three-day codesign agenda and methodology.

Duration	Activity	Description
Day 1: Shadowing and discovery		
60 min	CRH context and kickoff	Presentation by VA CRH program leadership covering network structure, telehealth & care modalities, mental health growth areas, and warm-handoff workflows.
45 min	Technology station tour	Guided tour of CRH-deployed telehealth carts and peripherals in the simulation room.
180 min	Shadowed sessions (four)	Two sessions centered on in-clinic telehealth cart delivery with a telehealth technician operating peripherals; two provider-workflow discussions including documentation and warm handoffs.
45 min	Group debrief	Themes and questions surfaced and carried into Day 2; all sessions audio recorded for thematic analysis.
Day 2: Full-day codesign workshop		
30 min	Introductions and icebreakers	Welcome, roles, and one-word check-in to surface expertise and build rapport.
30 min	Orientation and product overview	Workshop objectives, Immergo platform overview, and a brief mental health VA CRH landscape presentation.
45 min	Design warm-up & XR tutorial	Design thinking warm-up on identifying UX quality traits of user-favored technology, Meta Quest first-time user tutorial on Immergo, and hands-on time with the Immergo build and web application.
60 min	Platform exploration (SEQ tasks)	Seven semistructured usability tasks (Figure 2), each rated with the Single Ease Question.
75 min	Four-axis framework	Within-team Raise, Reduce, Eliminate, and Create ideation on sticky notes framed around mental health, warm handoffs, and whole health; ideas synthesized to a shared team board.
30 min	Within-team prioritization	Teams ranked ideas from most to least important on an importance prioritization worksheet.
30 min	Cross-team gallery voting	Gallery walk; each non-product participant received two voting stickers per other team to endorse the ideas they judged most important.
30 min	Recap and exit survey	Synthesis of top themes, postworkshop survey, one-word closeout.
Day 3: Debrief and action mapping		
60 min	Team readouts	Readouts of prioritized stickies and workshop artifacts with Immergo and VA research leads.
90 min	Action mapping	Findings organized into buckets: mental health use cases to prototype, evidence needed, deployment considerations, and collaboration routes; sticky-note proposals captured throughout.
30 min	Wrap-up	Owned action items and next steps; remaining postworkshop surveys collected.

Breaks omitted for brevity.

### 3.5 Materials and technology

The teams used the Immergo platform (application version v0.287) for Meta Quest OpenXR, with each workshop table equipped with at least two Meta Quest 3S headsets so participants could explore the VR application as needed. Laptops were provided to display VR views to the group so they could follow along and take notes. Because the Immergo application had not yet completed onboarding to the VA’s internal network at the time of this activity, evaluating the platform required a temporary, non-VA connectivity path. All sessions therefore ran over a dedicated commercial Wi-Fi hotspot (AT&T NETGEAR Nighthawk M7 Pro Mobile Hotspot 5G mmWave WiFi 7 with up to 64 device connections), a standard testing arrangement for preonboarding pilot activities of this kind. All participants were given Immergo care provider user accounts for codesign, enabling both VR and web application logins. When not using the VR headsets, the participants worked in Immergo’s web application, which affords appointment scheduling, avatar customization, camera-based full-body tracking, movement measure review, and exercise program assignment; the web application is also embedded inside the VR application, and participants explored a help page in the web application in parallel with completing the VR tutorial. The seven VR usability tasks are shown in Figure 2, and the corresponding web application features are shown in Figure 3.

**Figure 2:**
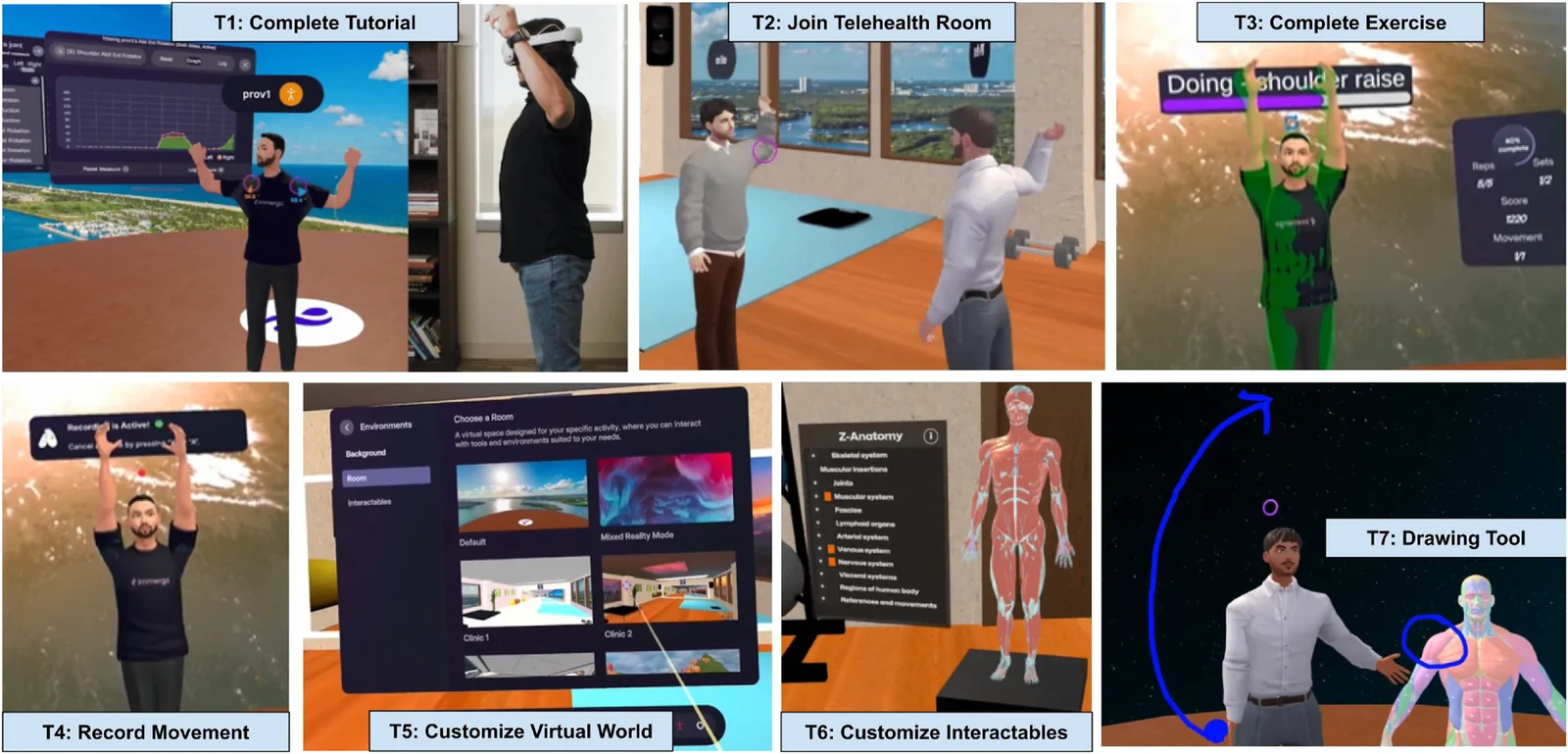
The seven VR usability tasks completed on the Immergo platform (v0.287). T1: completing the Immergo tutorial, which includes previewing controls, environment customization, a mock telehealth appointment, movement recording, exercise program creation, and a sample exercise program. T2: joining a telehealth room by creating a custom meeting room code and entering it as a full-body avatar. T3: completing an assigned exercise program with guided movement playback. T4: recording a movement and saving it with a name for later review. T5: customizing the virtual world by switching between 3D environments. T6: customizing interactables by selecting, placing, and manipulating 3D content in the room. T7: using the spatial drawing tool to draw and annotate in the shared 3D space.

**Figure 3:**
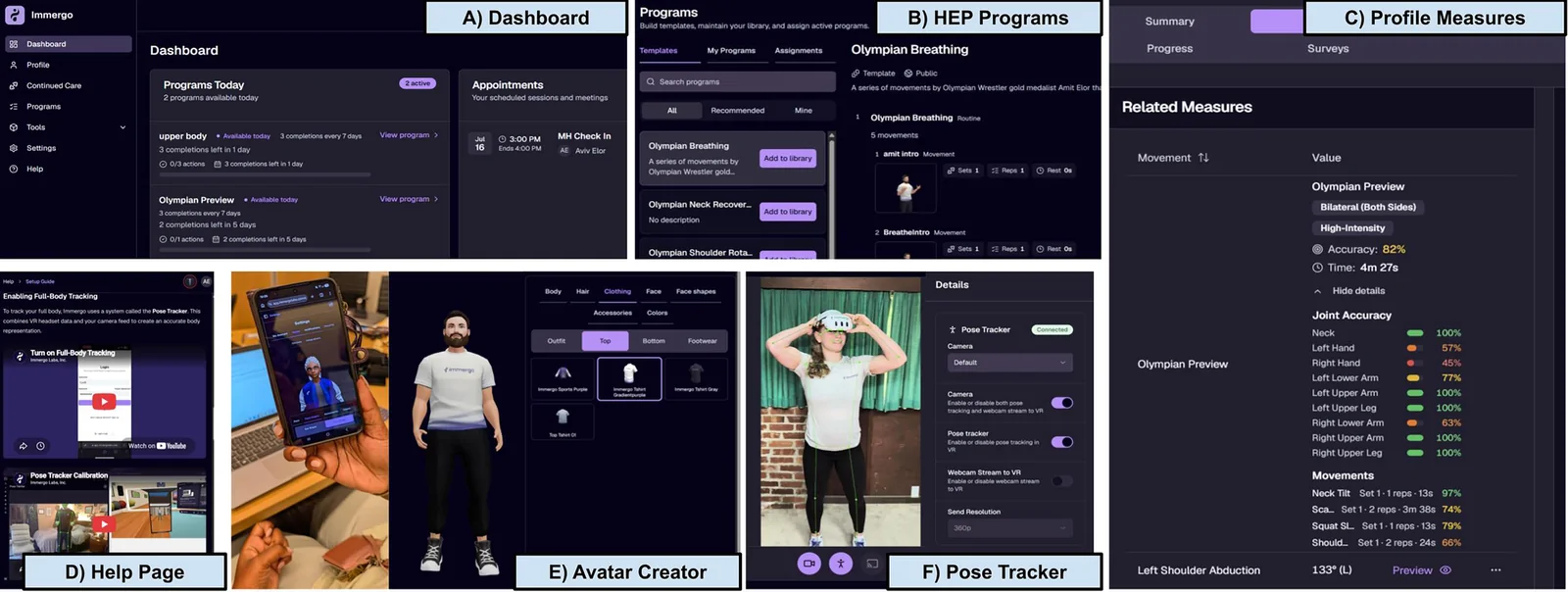
The Immergo companion web application features explored during the workshop, including (**A**) the dashboard page with telehealth appointment scheduling and home exercise program review, (**B**) the movement program library page for home exercise creation and assignment, (**C**) the profile page with session summaries and recent measure review, (**D**) the help page with written feature guides and videos, (**E**) the avatar creator page for personalizing the user's full-body avatar, and (**F**) the tools page with the full-body tracker webcam joint keypoint streaming tool. Panels (**A**)–(**E**) are also embedded in the VR app, whereas (**F**) streams data to the VR app for full-body tracking.

Printed materials included a usability task worksheet, a four-axis framework worksheet, and an importance prioritization worksheet, drafts of which are shared in the Appendix for reuse. Participants were also given sticky notes, pens, and voting stickers. Each non-product participant received two voting stickers per other team, to be used at the end of Day 2 for gallery voting, in which they could endorse up to two ideas they felt were most important from each of the other teams’ boards.

### 3.6 Data sources

Data collection spanned all 3 days and is reported by instrument, as follows:

- *Usability task worksheets.* Single Ease Question ratings for each of seven VR tasks from up to 21 raters per task (VA stakeholders), with free-text notes.
- *Four-axis worksheets and team boards.* In total, 164 sticky-note design ideas were generated across the five teams under the Raise, Reduce, Eliminate, and Create prompts.
- *Importance prioritization worksheets.* Within-team rankings of ideas from most to least important.
- *Cross-team dot votes.* In total, 188 voting-sticker endorsements were cast during the Day 2 codesign gallery walk.
- *Debrief sticky notes.* In total, 95 Day 3 debrief proposals were captured during team readouts and action mapping.
- *Discussion transcripts.* In total, 19 audio-recorded sessions were converted to text via speech-to-text: 6 Day 1 sessions (kickoff, context-setting discussion, and four shadowed sessions; approximately 53,000 words), 10 Day 2 sessions (workshop kickoff, per-team tutorial reflections and four-axis reflections, and wrap-up; approximately 48,000 words), and 3 Day 3 sessions (debrief and action mapping; approximately 21,000 words), totaling approximately 122,000 words.
- *Surveys.* A preworkshop survey (n=18) and a postworkshop survey (n=17) with structured items and open responses.

### 3.7 Quantitative analysis

Usability for each task was assessed using the SEQ, a validated single-item post-task measure (62), administered on a 5-point scale (1=very difficult,5=very easy). The postworkshop survey assessed workshop quality, perceived clinical relevance, alignment with participant expectations, and included a 0-to-10 recommendation item, scored as a Net Promoter Score (68). Prioritization was determined by idea-level vote counts. Each non-product participant could endorse up to two ideas on another team’s board. Not all participants used their full allotment, and Immergo product team members did not cast any voting stickers. Therefore, all 188 votes represent endorsements by VA stakeholders and embedded SMEs. Vote counts are a structured prioritization signal within this workshop, as votes could only attach to written ideas, and totals depend on idea phrasing and overlap. Within-team importance rankings from the prioritization worksheets were converted to percentiles (1.0=ranked most important on the team’s board). Because priority-bar placement varied across teams, percentiles are directly comparable within a team and provide indicative comparisons across teams. Immergo product team members also did not complete usability ratings. SEQ ratings were provided by VA stakeholders and SMEs, with the number of responses per task varying by completion. Consistent with prior codesign work, all quantitative measures were analyzed descriptively, with exact n values reported for each estimate. These results should be interpreted as formative design insights that contextualize the qualitative analysis, rather than as statistically generalizable outcomes (27).

### 3.8 Qualitative analysis

A reflexive thematic analysis (65, 66) was conducted across 19 speech-to-text discussion transcripts and workshop artifacts, including four-axis ideas, debrief stickies, usability notes, and open survey responses. Coding employed a 49-code, nine-domain codebook built deductively from the teams’ own board groupings and refined inductively across a full transcript reading, with revisions logged in the analysis worksheets. Transcripts were reviewed by the study team before any AI-assisted step; after the codebook was defined, Claude Opus 4.8 (Anthropic, CA, USA) recommended sentence-level code assignments with direct transcript evidence, each reviewed and corrected by a human rater against source transcripts, followed by a second round of thematic analysis. All analytic judgments remained with the investigators. Coding was performed by a single coder and peer-reviewed by the coauthors, with interpretive differences resolved by consensus and each theme searched for counter-examples beyond the postsurvey detractors. The coding author is an HCI researcher, principal investigator, and technology developer; VA and academic coauthors without commercial affiliation also reviewed the themes. To mitigate potential facilitation influence due to the inclusion of Immergo team members as facilitators, independent SMEs were embedded across the teams. The product team was excluded from usability ratings and gallery voting. Negative cases, including postsurvey detractors, were incorporated into the analysis and discussion. Given that the 3 days comprised different session types, the primary thematic structure is organized by day, with five categories per day, each further divided into key themes. Within each key theme, evidence signals were derived from distinct data sources and were not combined, as follows: *Said* (transcript units coded for the theme), *Proposed* (sticky-note ideas coded for the theme), *Voted* (voting stickers attached to the theme’s Day 2 ideas; stickers could only be placed on written ideas, not transcript turns, so Said and Voted are structurally independent), and *Prioritized* (within-team importance percentile of the theme’s ideas).

### 3.9 Ethical considerations

This activity was conducted as part of QI work and was reviewed by the Michael E. DeBakey VAMC (Houston) Research and Development Committee. QI is a common precedent for operations activities designed to improve VA programs and services (32). A Data Use Agreement between Immergo Labs and Houston VAMC IQuESt governed data sharing with data reviewed and audited by authors. No protected health information was collected, and participation in all surveys and instruments was voluntary.

## 4 Results

### 4.1 Overview of findings

This section is organized around three main findings aligned with the study questions. First (SQ1), Day 1 shadowing identified that key constraints for new modalities occur before headset use. Connectivity, device provisioning, workload, and governance already define telehealth mental healthcare for a workforce primarily serving veterans at home. Second (SQ2), perceived task ease was high across core embodied telehealth tasks for this group, which had telehealth experience but mixed VR exposure. The prioritization exercise revealed a divergence: therapeutic capability generated the most discussion, but minimalist defaults, onboarding, and embodiment received the most votes. Third (SQ3), Day 3 established the following initial development priorities: breathing and grounding, exposure, psychoeducation, movement for whole-health activities, and identity-centered embodiment, all designed for plug-and-play use. Deployment was proposed to begin with tablets in rural clinics with existing network infrastructure, or through approved home deployments, in line with workforce requirements and a QI-first approach. All quantitative results are reported descriptively as part of the formative design context.

### 4.2 Participants already deliver multimodal, home-reaching care (SQ1)

Presurvey respondents (17 of 21 VA stakeholders and one SME) reported experience in delivering care across clinic-based video telehealth, video into non-VA locations, telephone, and in-person visits, with telehealth constituting a mean of 81% (median 94%) of their care and the largest share (63%) reaching veterans at home rather than in a VA clinic room (Table 2). The cohort already uses both synchronous and asynchronous modalities, which influenced their evaluation of the platform’s live and asynchronous modes.

### 4.3 Day 1—shadowing: the current-state baseline (SQ1)

Day 1 was observational and generated transcript evidence. Table 4 summarizes the five current-state categories and key themes, described in detail below.

**Table 4:** Day 1 shadowing: current-state categories and key themes.

Category	Key theme	Details	Said
Current care pathways and handoffs (45)	Delivery configurations and warm handoffs	How telehealth actually moves a veteran between providers today	19
	Library, continuity, and provider onboarding	Provider requirements for knowledge acquisition and reuse during visits	17
	Team, group, and provider control	Care delivery involves coordinated team activities rather than individual clinicians	9
Infrastructure and device ceiling, already binding pre-VR (51)	Cart and room scarcity, EHR friction	Limited in-clinic telehealth carts and lack of integrated health records increase encounter complexity	27
	Network is the binding constraint	Facility connectivity is often unstable prior to VR implementation	12
	Devices and provisioning	Processes for ordering, issuing, returning, reusing, and managing device inventory	12
Veteran population: access, equity, whole-person needs (67)	Whole-person needs and current use cases	Pain, mental health, and whole health are addressed concurrently	37
	Accessibility, fit, and representation	The primary user base consists of older, disabled, and isolated veterans	16
	Rural reach and the digital divide	Access to care is determined by geographic distance and device availability	14
Safety, privacy, and governance in practice (30)	Crisis, distress, and contraindications	Providers maintain established safety protocols	14
	Security, credentialing, and trust	VA security standards and data trust requirements	12
	Data, disclosure, and liability	Patient-generated data present both opportunities and risks	4
Workforce capacity, training, and champions (41)	Champions, training, and the evidence problem	Adoption depends on key personnel and required evidence	20
	Partnership and collaboration routes	Institutional processes for pilot implementation	18
	Workload and production standards	New initiatives must compete with existing provider workloads	3

“Said” refers to coded transcript units. Day 1 was observational only; no ideas or votes were recorded.

*Veteran population: access, equity, and whole-person needs* was the largest category (67 mentioned/coded units). Providers reported that pain, mental health, and whole health are integrated in their caseloads, and not often treated as separate service lines. They described workarounds required by current 2D video, such as sharing worksheets and files during sessions and improvising exposure practice on a flat screen. Clinician presence was identified as the key factor in engagement during immersive use. Providers cited a previous chronic pain VR program with approximately 50% engagement when veterans used the headset alone, compared to nearly 100% when a clinician tracked progress. This estimate was repeatedly discussed as a reason to keep clinicians involved. Accessibility and representation were identified as practical constraints, including glasses fit, seated delivery, and serving older users. For veterans living far from facilities, telehealth is the primary means of access.

*Infrastructure and device ceiling* (51 units) binds before any headset is added. Unintegrated records increase workload for every encounter. Providers must navigate both the federal electronic health record and legacy systems, documenting the same findings twice. Telehealth carts and clinic rooms are scarce and tracked separately; connectivity often fails inside the facility; and device provisioning is a separate workflow involving ordering, issuing, returning, and reusing hardware. One participant captured the static-content problem with currently deployed VR devices: veterans “will be bowling for life until” the software is upgraded, because updating the VR headset fleet’s content is itself a governed process.

*Current care pathways and handoffs* (45 units) described how a veteran actually moves between providers today. Warm handoffs are an operational strength and the preferred approach, delivered same-day across multiple communication channels. Care is a team activity involving schedulers, telehealth technicians, and providers. The need for providers to learn, reuse, and maintain continuity across visits creates a hidden cost for any new modality, due to the library and onboarding burden of each tool.

*Workforce capacity, training, and champions* (41 units) surfaced the adoption calculus: anything new competes with a full panel, adoption travels through named internal champions, and providers articulated the evidence expectations that a new modality must meet before it earns clinic time. Participants began considering partnership and collaboration routes on Day 1, anticipating the action mapping on Day 3.

*Safety, privacy, and governance in practice* (30 units) documented the floor providers already operate on, as follows: crisis pathways, contraindication reasoning applied per veteran rather than per diagnosis (a seizure history, one provider noted, warrants caution rather than categorical exclusion from VR), the VA security and credentialing posture as the first barrier any vendor meets, and caution that patient-generated data cuts both ways once it must be memorialized in the chart.

### 4.4 Day 2—usability evaluation

Participants completed seven usability tasks on the Immergo platform, each rated using the Single Ease Question with 15–19 raters per task (results are shown in Table 5). Ratings were high overall, with the strongest results on the workflows most central to embodied telehealth, including customizing the virtual world (M=4.84±0.37), completing the tutorial (M=4.50±0.71), joining a telehealth room (M=4.47±0.84), and recording a movement (M=4.35±0.86). These results suggest that, for this telehealth-experienced but largely mixed VR experience provider cohort, the core loop of entering a shared embodied session, personalizing the environment, and capturing movement was rated as easy to complete under the workshop conditions, consistent with the environment personalization finding from our prior rehabilitation codesign (27). Friction concentrated in the newest tooling, customizing interactables (M=4.13±0.92), and the drawing tool (M=4.12±1.26), where modes and actions were not fully covered in Immergo’s interactive tutorials or leaned more heavily on controller-based inputs.

**Table 5:** Day 2 Single Ease Question ratings by task (1=very difficult,5=very easy), ordered by mean.

Task	*M* ± SD	N	Feedback summary
Customize the virtual world (T5)	4.84±0.37	19	Most positive; requests for more environment options
Complete the Immergo tutorial (T1)	4.50±0.71	18	Wanted more hands-on customization and expanded controller + hand tracking orientation
Join a telehealth room (T2)	4.47±0.84	19	Easy once connected; friction on Wi-Fi hotspots
Record a movement (T4)	4.35±0.86	17	Opportunity to expand start and stop cues, replay fidelity
Complete an exercise program (T3)	4.28±0.75	18	Wanted clearer movement-tab navigation, avatar alignment, target and depth cues
Customize interactables (T6)	4.13±0.92	15	Discoverability of moving objects; rotation, repositioning, and actions initially unclear for VR non-experts
Use the drawing tool (T7)	4.12±1.26	16	Brush, eraser, and color modes and quick-menu dismissal were less intuitive as they were mapped to controller buttons when wrist menu was not utilized

Ratings were completed by VA stakeholders and SMEs (Immergo product team members did not rate tasks); per-task N varies with completion.

Free-text task notes reinforced the following three recurring friction patterns: discoverability of features, an onboarding and tutorial framing gap, and control-mapping opacity. Network and connectivity interruptions were also the most frequently noted impediment overall, and occurred inside a VA facility with technical staff present, as the VR platform was not yet deployed on the VA’s internal network and ran over Wi-Fi hotspots for this study, foreshadowing the deployment findings of Section 4.6. Roughly three-quarters of respondents did not provide free-text feedback, and a quarter of those who did reported no difficulty at all, with several notes being additional feature requests rather than problems. Notably, the two lowest-rated tasks were also the two least covered by the tutorial in this build: the drawing tool’s brush, eraser, and color modes were mapped to controller buttons with wrist menus and alternative activation not yet included in the tutorial, and moving, rotating, and repositioning interactables were likewise untutored in Immergo v0.287 where the written text guides in the web application may not have been retained. The friction, therefore, reads at least in part as an onboarding/tutorial-coverage finding rather than as inherent task complexity, reinforcing the onboarding priority that dominated the four-axis endorsement.

### 4.5 Day 2—four-axis ideation and prioritization (SQ2)

The four-axis exercise generated 164 ideas and 188 gallery votes, all submitted by VA participants and SMEs. The distribution across axes is skewed, as follows: *Create* dominated generation with 84 of 164 ideas (51.2%) and 106 of 188 votes (56.4%), as expected for an exercise framed around extending the platform into mental health; *Raise* followed with 52 ideas (31.7%) and 54 votes (28.7%); *Reduce* drew votes above its idea share (16 ideas, 9.8%, against 20 votes, 10.6%); and *Eliminate* was smallest (12 ideas, eight votes). Approximately two-thirds of all ideas received no votes, resulting in concentrated endorsement for a limited subset of ideas.

To account for every idea rather than only the most-voted, we consolidated the teams’ own board groupings into 10 idea groupings spanning all 164 ideas and 188 votes (Table 6). The axis distribution paired with the groupings shows where generation and endorsement diverged. Personalization, identity, and representation attracted both the most ideas (30) and the most votes (46); onboarding, tutorials, and guidance converted 20 ideas into 43 votes, the second-highest endorsement density; and ease of use, minimalist defaults, and cognitive load drew 34 votes on 27 ideas even though the exercise asked participants to imagine a new capability. By contrast, environments and interactables, engagement and rewards, and VA system integration generated 34 ideas among them, but only 12 votes, frequently proposed but rarely endorsed for a first build.

**Table 6:** All 164 four-axis ideas consolidated into 10 idea groupings (normalized from the teams’ own board groupings), with idea counts per prompt (Create/Raise/Reduce/Eliminate), total gallery votes, and the most endorsed ideas per grouping (votes in parentheses).

Idea grouping	Ideas (C/R/Rd/E)	Votes	Most endorsed ideas
Personalization, identity, and representation	30 (16/12/1/1)	46	Personalized environments with full 360 views and disability representation (9); avatar badges, stripes, and stars for service area and rank (8); representation of seated and altered anatomy (4); military-themed avatar customization (4)
Onboarding, tutorials, and guidance	20 (7/11/2/0)	43	Very clear onboarding workflow for patient and clinician (10); guided AI assistance with just-in-time tutorials (8); clear, easy-to-follow tutorials throughout (7); immersive video explaining treatment and use (3)
Mental health and whole health content	29 (21/4/2/2)	38	Remove on-screen range-of-motion numbers for mental health sessions (7); behavioral activation with goal-setting and in-VR practice (6); searchable library by patient diagnosis and characteristics (5); de-escalation experiences for hyperarousal (4)
Ease of use, minimalist defaults, and cognitive load	27 (7/8/5/7)	34	Ensure client UI is simple (12); headset ready out of the box with reduced clicks (10); reduce provider clicks to navigate menus (5)
Environments and interactables	19 (14/4/1/0)	12	Practical life-skills environments such as stores, bars, streets, and schools (2); phobia-safe minimalist default environment (2); display images and diagrams to support instruction (2); varied ambient and stressor sounds (2)
Provider tools and functional workflow	5 (5/0/0/0)	9	EHR integration and single sign-on for the clinician web application (8); whiteboard with savable illustrations (1)
Connection, groups, and team-based care	12 (2/10/0/0)	4	Veterans joining group sessions together (4)
Safety and governance	7 (4/1/0/2)	2	Preventative measures for fall risk and collision (2)
VA system integration, access, and equipment	9 (3/1/5/0)	0	No ideas endorsed
Engagement, rewards, and social play	6 (5/1/0/0)	0	No ideas endorsed
Total	164 (84/52/16/12)	188	

Rows ordered by votes.

Each axis exhibited a distinct pattern of responses.

Within the *Create* axis (84 ideas, 106 votes), the most highly endorsed ideas combined identity and simplicity. These included highly personalized virtual environments with 360-degree views and disability representation (nine votes); a clear onboarding workflow for both patients and clinicians (10 votes); immediate headset readiness with fewer required actions (10 votes); electronic health record (EHR) integration with single sign-on for the clinician web application (eight votes); and avatar badges, stripes, and stars to indicate service area and rank as conversation starters (eight votes). Endorsed *Create* ideas related to clinical content included behavioral activation for depression with individualized goal-setting and in-VR practice (six votes), experiences designed to de-escalate hyperarousal following nightmares (four votes), and microphone-tracked breathing synchronization (four votes).

Within the *Raise* axis (52 ideas, 54 votes), participants emphasized the need for enhanced guidance and findability. Key proposals included increased guided artificial intelligence (AI) assistance with specialized just-in-time tutorials (eight votes), clear and accessible tutorials throughout the user experience (seven votes), a searchable library of exercises and interactables organized by patient diagnosis and characteristics (five votes), enabling veterans to join group sessions together (four votes), and expanded hover tooltips to explain each control (four votes).

The *Reduce* axis (16 ideas, 20 votes) included the workshop’s most highly endorsed idea: minimalist defaults for user interfaces (12 votes from four teams). Additional priorities were reducing the number of provider interactions required to navigate menus (five votes) and implementing a phobia-safe minimalist default environment (two votes).

The *Eliminate* axis (12 ideas, eight votes) was led by proposals to remove on-screen range-of-motion numbers, which were deemed unnecessary for mental health sessions (seven votes, endorsed by all five teams), and to eliminate movement-care instrumentation, considered clinically irrelevant in this context. Independent teams consistently prioritized minimalist defaults and onboarding across all axes, mirroring the patterns observed in usability friction analyses.

Table 7 summarizes the five Day 2 categories and their key themes, with all four axes represented separately. Figure 4 illustrates the divergence between discussion and voting at the key-theme level. Therapeutic capability and use cases generated the most extensive discussion (169 Said units), covering topics such as anxiety and breathing content; exposure practice; whole-health movement; and the care-model components of groups, homework, and handoffs. However, these topics received only 31 votes. Usability, onboarding, and the preference for minimalist defaults accounted for 107 Said units and 57 votes from only 18 ideas, representing the highest endorsement density in the workshop. Embodiment, avatar identity, and environment ranked second in votes (46), with key themes including personalized environments, veteran identity markers, and the use of nonverbal channels as clinical signals. Participants also emphasized that the treatment space should remain distinct from a game environment. Considerations regarding safety, deployment, and adoption re-emerged during the ideation process (75 Said units), while accessibility and representation remained distinct discussion categories.

**Table 7:** Day 2 categories and key themes with source-separated signals.

Category	Key theme	Details	Said	Prop.	Voted	Priority
Usability, onboarding, and the preference for minimalist defaults (107/18/57)	Onboarding on rails and preview	Show a clinician what this is in the first minute	39	1	3	1.00
	Control-scheme opacity and input alternatives	Buttons, hands-free, and voice as the friction surface	39	7	12	0.76
	Minimalist defaults	The room optimized by removing, not adding	23	9	34	0.72
	Just-in-time and AI-assisted guidance	Help that appears at the moment of need	6	1	8	1.00
Embodiment, avatar identity, and environment (65/32/46)	Environment personalization and cultural fit	Nature, patriotic, neutral, and meaningful objects; keeping the therapeutic frame distinct from a game	39	22	30	0.68
	Veteran identity markers and representation	Rank, uniforms, and disability, seen on the avatar	15	2	8	0.61
	Non-verbal channel and presence	Eye contact and expression as clinical signal	11	8	8	0.75
Therapeutic capability and use cases (169/29/31)	Groups, homework, handoffs, education, engagement	The care-model machinery around a session	79	13	15	0.53
	Whole-health movements	Guided movements such as tai chi and yoga with real-time feedback	36	8	2	0.49
	Anxiety, breathing, meditation, and sleep	The easy-win mental health content	34	5	6	0.67
Reality checks: safety, deployment, adoption (75/11/20)	Exposure and in-vivo practice	Graded, safe rehearsal of feared situations	20	3	8	0.44
	Network, devices, EHR, security	The deployment ceiling resurfacing mid-ideation	30	7	17	0.75
	Adoption: champions, training, and evidence	Buy-in and legitimacy raised while ideating	29	1	–	0.89
	Disclosure, data, AI scope, and trust	Who sees the data and where automation stops	9	–	–	–
	Physical safety, crisis, and contraindication	Design ideas that trip the safety floor	7	3	3	0.89
Accessibility and representation (29/5/6)	Accessibility and physical fit	Glasses, seated use, big buttons, users new to technology	17	3	6	0.78
	Connection, avoidance, and fit	Loneliness answered, avoidance not enabled, VR is needs dependent	9	2	–	0.11
	Rural reach and the digital divide	Design that survives a bad connection and no setup	3	–	–	–

Said, coded transcript units; Prop., four-axis ideas; Voted, gallery votes on those ideas; Priority, mean within-team importance percentile of the theme’s ideas (1.0=ranked most important).

**Figure 4:**
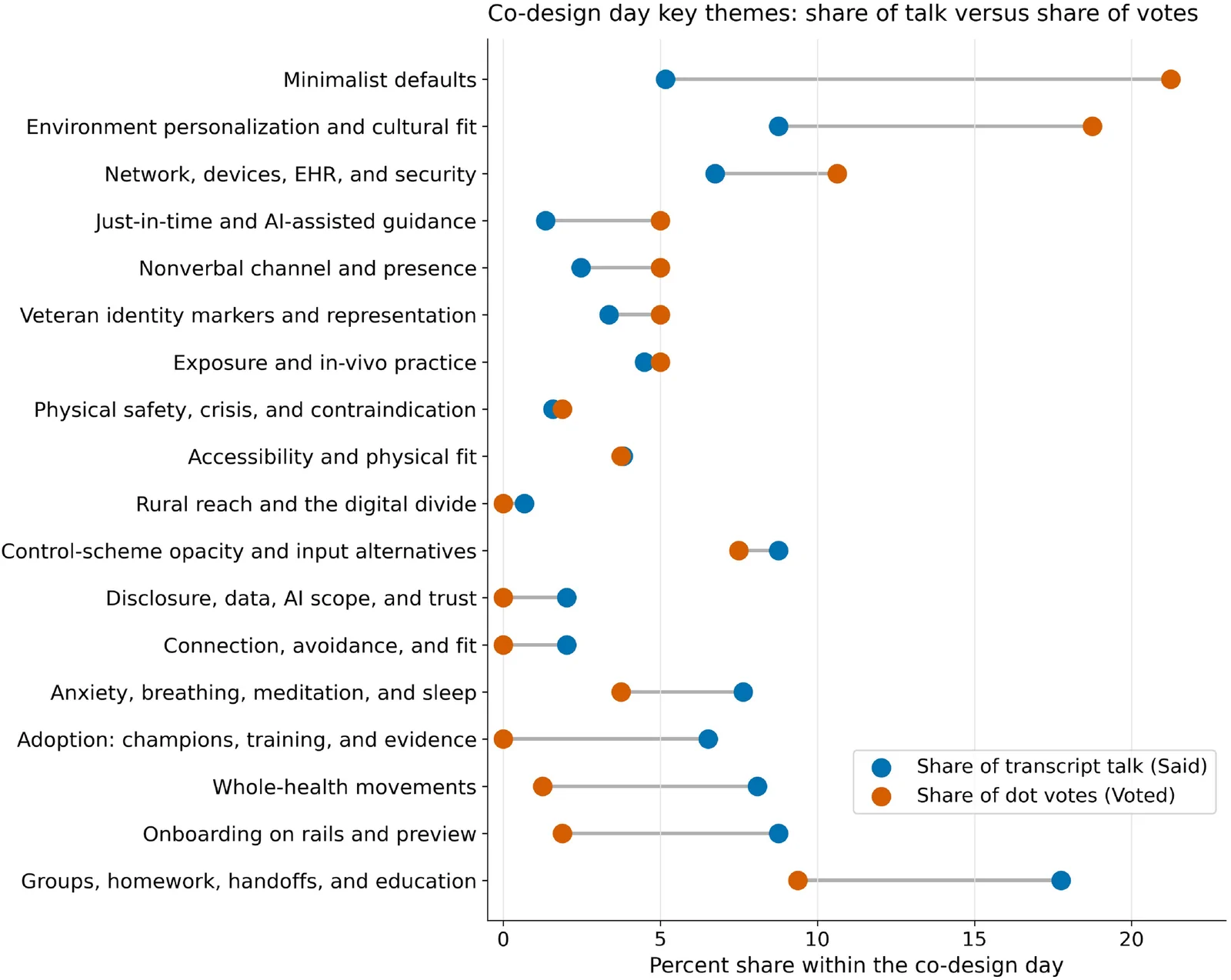
Day 2 divergence between share of transcript talk (Said, blue) and share of gallery votes (Voted, orange) per key theme, sorted by gap. Minimalist defaults, environment personalization, and identity representation were voted above their share of talk; therapeutic capability machinery, whole-health movement, onboarding discussion, and adoption were discussed above their votes. Because a voting sticker can only be attached to a written idea, never to a transcript turn, the two shares are structurally independent, and their divergence should be interpreted as a difference in how ideas were expressed and selected. Note that onboarding drew heavy discussion but few dedicated stickies, so its endorsement was carried by the top-voted onboarding ideas within the onboarding and minimalist-defaults groupings (Table 6).

Within *usability, onboarding, and the preference for minimalist defaults*, onboarding and control-scheme opacity carried the discussion (39 Said units each) while minimalist defaults carried the votes (34 of the category’s 57). Participants asked for input alternatives on behalf of their veterans: “for my population, [we need] more hands-free functionality, whether that’s voice or eye-gaze tracking,” as one participant put it, so that the controller does not exclude veterans with fine-motor control issues. The preference for minimalist defaults was similarly explicit. In describing the ideal asynchronous setup, one participant summarized: “all you do is tell them to set the camera on the table and put the headset on… something super simple [to get the equipment ready].”

Within *embodiment, avatar identity, and environment*, environment personalization was the dominant proposal surface (22 of the category’s 32 ideas), spanning nature, patriotic, neutral, and personally meaningful content; one participant reflected, “I liked all the adaptations and being able to customize the room [virtual environments],” while wishing for a whiteboard interactable to write on mid-session beyond free annotation in 3D. Providers conceptualized identity markers as clinical conversation starters rather than mere decoration. For example, an avatar displaying deployment history “opens up an immediate dialog… it looks like you deployed for 3 years, where were you?”

Within *therapeutic capability and use cases*, the care-model machinery of groups, homework, handoffs, education, and engagement was the single most discussed key theme of the entire workshop (79 Said units), covering how an embodied room would host group sessions, how homework travels between visits, and how a warm handoff could move a veteran into the room live. Whole-health movements (36 units) surfaced tai chi, yoga, and guided movement as content this platform is already positioned to deliver; anxiety, breathing, meditation, and sleep (34 units) were repeatedly named as the easy-win content to lead with; and exposure discussion emphasized graded, provider-adjustable rehearsal of feared situations rather than intensity.

Within *reality checks*, the deployment ceiling resurfaced mid-ideation (30 Said units, 17 votes), with even workshop facilitation made contingent on connectivity, and adoption concerns (champions, training, evidence) were raised while ideating (29 units) yet never converted into votable stickies.

Within *accessibility and representation*, physical fit concerns (glasses, seated use, large targets, users new to technology) drew steady discussion and votes. The connection theme cut both ways: “our veterans have so much loneliness,” one participant observed, arguing that a product that can cross that distance would matter, while others cautioned that immersion must not become an avoidance channel and that VR is needs-dependent for veterans.

### 4.6 Day 3—debrief: what to build first and deployment paths (SQ3)

Day 3 shifted from what the platform could do to what deployment requires. The half-day is summarized by transcript themes and sticky-note proposals (Table 8), along with 95 action-mapping proposals across four categories described below.

**Table 8:** Day 3 debrief categories and key themes.

Category (Said/Prop)	Key theme	Details	Said	Prop.
What to build first: prioritized capabilities (160/30)	Embodiment, environment, and identity	The representation and environment requirements	54	9
	Mental health capabilities to lead with	Breathing, exposure, education, movement, and biometrics	47	13
	Care model and delivery	Session configurations, groups, handoffs, homework, and library	33	6
	Simplicity, onboarding, and engagement	Plug and play stated as non-negotiable	26	2
The deployment ceiling and how to clear it (46/8)	Anchor to existing infrastructure	Ride the in-clinic tablets and the telehealth cart to support VR headset usage	22	1
	Devices, provisioning, MDM, and records	Fleet management and record integration to solve	21	5
	Network as the binding constraint	Supporting compatibility	3	2
Workforce, evidence, and legitimacy (36/11)	QI-first evidence and the perception problem	Start with QI; user stories, engagement, and satisfaction	15	6
	Training, workload, and clinician tools	Fit the clinician workflow, not only the patient	11	1
	Champions and the consult chain	Named people who carry it into the system	10	4
Governance, safety, and the limits of automation (16/1)	AI guardrails and scope of practice	Standard information yes, clinical decisions no	9	–
	Crisis, disclosure, liability, and physical safety	The disclaimers and pathways deployment requires	7	1
	Trust and the black box	Data trust and credentialing as adoption gates	–	–
Collaboration and partnership routes (39/10)	Academic and clinical partnerships	Capstones, IRB amendments, and VA Immersive	21	9
	Rural reach and equity as a use case	Isolation and accessibility as where to prove value	14	1
	The multivendor ecosystem	Vendor agnosticism as a standing VA constraint	4	–

Said, coded transcript units; Prop., debrief sticky-note proposals. No voting was conducted on Day 3.

*What to build first* dominated the discussion (160 Said units, 30 proposals). Participants converged on the mental health capabilities to lead with, as follows: breathing and grounding, graded exposure, psychoeducation, whole-health movement (“even Tai Chi… you can still do some of it” seated or upper-body only, one participant noted), and biometrics presented on the provider’s terms, including open questions about when and how a veteran should see or share their own physiological data in a group setting such as affecting avatar visual states (with data hidden by default). Embodiment, environment, and identity requirements were the most discussed (54 units), reinforcing Day 2’s recommendations as build requirements. Care-model requirements included session configurations, group management, homework, handoffs, and an exercise content library. Simplicity and plug-and-play functionality were identified as essential.

*The deployment ceiling* category contained the workshop’s most quotable strategic guidance: anchor to existing infrastructure rather than introduce a new managed device. As one participant reasoned, “building the [tablet support] clinical interface is something that [should be] relatively simple to do because you already have something going,” riding the tablets currently used for the provider to assist the veteran with remote management is something the network can already support. Device provisioning, mobile device management (MDM), and record integration were identified as manageable deployment challenges. *Workforce, evidence, and legitimacy* named provider champions and the consult chain (“critical to get institutional buy-in, but also to build the confidence for the people that are using this”), and a QI-first evidence path; participants pressed for the why behind each tool, warning that “this is cool tech” alone is not an adoption argument. *Governance* stayed consistent in stance: automation may deliver standard information, but clinical decisions stay with the provider, and crisis pathways, disclosure rules, and liability clarity are deployment preconditions, a stance participants grounded in their clinical experience with veteran suicide risk. *Collaboration routes* mapped academic and industry partnerships, QI or IRB-approved research, VA Immersive as the institutional channel, and rural reach and isolation as the equity use case in which to prove value first.

The 95 action-mapping proposals were grouped into the following four categories: deployment (31), mental health use cases (29), collaboration routes (19), and evidence and research questions (16). This distribution reflects the shift from capability to deployment. Mental health use-case proposals included breathing and grounding content, graded exposure with clinician-adjustable variables, behavioral activation, group session formats, and psychoeducation. Deployment proposals focused on pilot mechanics such as small multiweek pilots, home headset trials, and tablet-based provider control, with removal of unnecessary in-clinic telehealth carts. Evidence proposals requested provider and veteran testimonials, satisfaction, and usage measures. Collaboration proposals included university partnership grants, capstone projects, QI or IRB-approved research, and ongoing advisory involvement from workshop participants.

A closing gut-check enumerated deployment enablers and constraints (Table 9). The enablers read as an equity-oriented design checklist, as follows: reliable connectivity with fast joins, plug-and-play onboarding, vendor-agnostic distribution, documentation reaching the record, a clinician in the loop, tablet-paired headsets in clinic, and a defined support pathway. The constraints mirror them, with two notable additions: anything perceived as AI making clinical judgments, and leading adoption conversations with use cases providers flagged as carrying an existing evidence-perception bias.

**Table 9:** Day 3 deployment enablers and constraints identified by participants.

Enablers (must be true)	Constraints (what would stop it)
Reliable Wi-Fi; connect in under 5 minutes	Connectivity failure; veterans give up fast
Plug-and-play, under-an-hour onboarding	Onboarding that turns providers into technical support
Vendor-agnostic application distribution into the VA	Locking veterans to a single vendor
Integrate into the EHR; for piloting, simple data export is the minimum	Innovation that never reaches the record
Safety guardrails with a clinician in the loop	Anything perceived as AI making clinical judgments
Tablet-paired headset in clinic; help desk and service levels	Cart-bound delivery; no support path
QI-first evidence and patient-story video	Leading with use cases that carry an existing evidence-perception bias

### 4.7 Exit-survey sentiment

Postworkshop respondents (n=17, 77% of VA stakeholders and one SME) rated the workshop near the ceiling while rating the hands-on platform experience more moderately (Table 10). The lowest-rated aspects, hands-on platform experience and perceived clinical relevance, align with the friction observed in newer tooling and with the platform’s rehabilitation-first content at the time of the workshop. The Net Promoter Score was +41, with only three detractors citing specific structural mismatches between the platform and their practice; their responses are carried forward as negative cases in Section 5. A recurring open-response observation was that clinicians formed realistic visions of use quickly and found VR easier than expected.

**Table 10:** Postworkshop survey results (n=17; 16 VA stakeholders and 1 SME).

Measure	Result
Overall satisfaction (1–5)	4.82±0.53 (15 of 17 very satisfied)
Engagement and feedback contribution (1–4)	4.00±0.00 and 3.94±0.24; engagement rated excellent by all 17
Organization and flow/clarity of objectives (1–4)	3.82±0.53 and 3.59±0.71
Hands-on platform experience (1–4)	3.35±0.79 (lowest aspect; three rated fair)
Perceived clinical relevance (1–4)	3.29±0.99 (10 highly relevant; 1 not relevant)
Platform user-friendliness (1–5)	3.76±0.97
Met functionality expectations (1–4, n=14)	3.00±0.96
Would recommend (0–10)	8.2±2.36; NPS +41 (10 promoters, 4 passives, 3 detractors)
Follow-up interest/early-access opt-in	88%/76%

### 4.8 Cross-day synthesis: six throughlines

Read across the standalone days, six pooled reflexive themes connect the categories. The usability tax and the preference for minimalist defaults are the strongest themes by vote concentration: when prompted to create, the room endorsed removal, minimalist defaults, and onboarding, drawing 63 Day 2 votes on 224 coded units, the strongest endorsement density in the workshop. New shapes of care around the headset is the most voluminous and the least endorsed per unit (345 units, 31 votes), which is itself a signal: exciting to discuss, but not what the room would build first. Embodiment as the argument and the hazard (206 units, 38 votes) pairs the reasons this is not video (identity, non-verbal signals, and environment) with its sharpest risks (triggering environments, blurred treatment frames, and enablement of avoidance). Fitting into the workforce (148 units, zero votes) carries the highest within-team priority of any throughline, yet drew no endorsement because workforce fit was never turned into a votable four-axis idea, a priority the voting mechanism structurally could not see. The infrastructure ceiling (161 units, 17 votes) is pervasive across all days and sources but rarely voted on, and governing risk and the limits of automation (59 units, three votes) is the smallest in volume and the most consistent in stance: standard information, yes; clinical decisions, no. Equity did not form a seventh theme; it cross-cuts three of the six (accessibility within minimalist defaults, disability representation and loneliness within embodiment, and the digital divide and device scarcity within infrastructure) across 156 units spanning all sources and days.

## 5 Discussion

This study examined how an embodied XR movement care platform should extend into mental health and whole health within a national telehealth program serving underserved veterans, convened at a single host site (VISN 20 CRH Boise) with attending stakeholders drawn from several participating regional hubs. A central finding across all 3 days is that implementation constraints surfaced alongside, and sometimes ahead of, new XR capabilities. For SQ1 (current state), the constraints governing any new modality precede the headset: a home-reaching, telehealth-saturated workforce operates under connectivity, provisioning, record, workload, and governance ceilings that vary by team and setting, as documented on Day 1 (Sections 4.2 and 4.3) and interpreted in Section 5.4. For SQ2 (design priorities), talk and endorsement diverged: therapeutic capability dominated the discussion, while minimalist defaults, onboarding, and identity-centered embodiment dominated the votes (Section 4.5), a pattern interpreted in Sections 5.2 and 5.3. For SQ3 (build and deployment paths), the debrief converged on leading with breathing and grounding, graded exposure, psychoeducation, and whole-health movement, anchored to the tablet and cart infrastructure the network already runs when in clinic or through veteran home-approved deployment pilots, and on workforce terms with a QI-first evidence path and role-specific deployment gates (Section 4.6), interpreted in Sections 5.4 and 5.5. The following subsections develop these answers, revisit the design principles established in our physical rehabilitation codesigns (27, 28) in light of evidence from this cohort, and consolidate new considerations for mental health and whole health.

### 5.1 Minimalist defaults with expert customization: a durable throughline

Across all three workshops in this series, stakeholders converged on the same foundation: a minimal default experience that experts can progressively customize based on user needs and preferences. Private practice stakeholders voted minimalist interface navigation their top priority (27); this CRH cohort, explicitly prompted to create new capabilities, still concentrated its endorsement on minimalist defaults, a clear onboarding workflow, and out-of-the-box readiness as the three most-voted ideas across independent teams. Notably, these providers did not evaluate client-facing profiles or caseloads for this workshop; their endorsement reflects a belief in minimalism with expert customization that echoes the progressive complexity with the minimalist defaults principle of the rehabilitation codesign (27), now remotivated. Where private practice framed simplicity around patient novice-ness, this cohort framed it around workforce cognitive load and home deployment. For a workforce that is telehealth-saturated but uneven in VR experience, with half at never-to-limited exposure, every interface element is a tax paid before any therapeutic value is delivered (37, 38), and provider inexperience with telehealth VR is the norm rather than the exception for now (25). The practical implication is that, in a national telehealth network, progressive complexity should start closer to zero: the platform is defined less by its richest capability than by the fewest steps between putting on a headset and starting a session.

### 5.2 Embodiment as a key differentiator for embodied telehealth

Embodiment has been the differentiating thread of this codesign series from its start. In physical rehabilitation, embodied guidance with real-time feedback enabled the remote movement correction that 2D video lacks, where clinicians reported feeling like life coaches (21, 27). This cohort extended the same throughline into mental health: participants articulated embodiment as the reason this platform is not just another video codec, naming the non-verbal channel (posture, eye contact, and expression) as a clinical signal, environments that mean something to the veteran, and self-representation that carries identity, including rank, age, body type, disability, and glasses. Embodiment carried the second-largest vote share of any category, and the top Create idea paired personalized environments with disability representation. This aligns with the mechanism literature, in which embodiment can intensify emotional engagement (24, 35) and avatar identity shapes behavior and therapeutic effect (44, 46, 47).

What mental health adds to the throughline is the hazard side. The same participants who argued for embodiment immediately paired it with risks that physical rehabilitation never surfaced with this sharpness: environments that trigger rather than soothe, a treatment frame that blurs into a game, and immersion that enables avoidance rather than graded approach. For embodied telehealth, then, representation is simultaneously an engagement feature, an equity feature, and a clinical safety surface, and it should ship with paired safeguards (Table 11). Identity representation itself should be opt-in and veteran-controlled: service salience such as rank, unit, and deployment history is a therapeutic opener for some veterans and unwelcome in a therapy space for others, so the veteran, not the platform or the provider alone, decides what their avatar discloses. Note that all of these remain stakeholder-identified design directions; no therapeutic outcomes or safety measures were evaluated here, and exposure work in particular would remain provider-governed.

**Table 11:** Design principles from the physical rehabilitation codesign (27) revisited against this mental health and whole health stakeholder cohort, with extensions and new principles.

Prior principle	Evidence in this cohort	Extension for MH and whole health integrated care
1. Embodied guidance with real-time feedback	Non-verbal communication and presence were prioritized beyond verbal interaction; posture, gaze, and expression identified as clinical signals	Guidance now includes both movement correction and emotional presence
2. Progressive complexity with minimalist defaults	Top ideas focused on simplification, onboarding, and immediate usability	Minimalist defaults address workforce cognitive load and support home use
3. Adaptive accessibility through multimodal input	Glasses compatibility, seated use, large targets, and hands-free or voice options identified	Accessibility extends to representation: avatar body types spanning older, disabled, and seated veterans as an access requirement
4. Clinical authenticity via domain-specific assessments	Providers use contextual measures; personalized environments with disability representation were the top idea; identity markers supported by all teams	Authenticity broadens to identity-authentic representation and selective assessment: avatar, environment, and measurement match the clinical moment, safeguarded by opt-in veteran-controlled identity, provider-curated environment libraries, conservative defaults, and explicit treatment-frame cues
5. Biomechanical precision for trust and safety	Trust focused on crisis pathways, disclosure rules, and data governance	Trust extends from measurement precision to governance precision: settled crisis, disclosure, and liability pathways as deployment preconditions
6. Contextual onboarding for therapeutic competency	Onboarding was highly endorsed; onboarding under 1 hour required for deployment	Onboarding is an access and equity factor for both providers and veterans, with guided onboarding as the default
7. Infrastructure-anchored equitable deployment (new)	Use tablets or web interfaces for provider support; maintain vendor neutrality	Integrate with existing device fleets and management systems. Ensure the system works on the lowest-resourced connections
8. Information-only automation with the clinician in the loop (new)	Participants held that automation can provide standard information, but clinical decisions must remain with the provider. Any AI making clinical judgments is a deployment blocker	Limit automation to guidance, documentation, and standard information. Keep assessment and clinical decisions with the provider. Design the system to give clinicians more information upon request, not reduce their presence

### 5.3 Accessibility, infrastructure, and equity-fit deployment

Our Houston VA rehabilitation workshop found that integrated-network providers elevate accessibility and adaptation to a foundational requirement (28), and this cohort reproduced that pattern in two registers. The first area is adaptive accessibility for veterans with disabilities, including properly fitting glasses, options for seated use, large interface targets, and hands-free or voice-input controls. The second area concerns infrastructure. The most frequently noted usability impediment was the network, which failed inside a VA facility with technical staff standing by; participants generalized the point without prompting, reasoning that if connectivity breaks down in the building, it breaks down even harder in the rural homes CRH exists to reach (8, 10). Streaming positional keypoint data can require significantly less bandwidth than streaming full video, making it more viable for the satellite and low-bandwidth connections common in rural veteran homes. Home internet quality varies widely: satellite, broadband, and fiber connections each have distinct bandwidth, latency, and reliability profiles that can affect user experience relative to enterprise-grade connections (8, 10). Day 3’s strategic consensus, anchor to a tablet or a web control interface for additional provider assistance that veterans and telehealth technicians already manage when in rural clinics or follow existing approved at-home deployments, is the stakeholder-articulated version of what digital health equity frameworks prescribe: design that survives a bad connection, minimizes new device burden, and meets users on infrastructure they already trust (5, 6, 56). The resulting design rule for equitable XR telehealth is that the platform must degrade gracefully to the connectivity and device fleet of its least-resourced user, or it will silently exclude the rural veterans and veterans with disabilities the program seeks to support.

### 5.4 Workforce fit, evidence, and governance as deployment gates

Participants emphasized that anything new competes with a full panel; adoption travels through named champions and the consult chain; and clinicians expect evidence of efficacy prior to adoption (39, 40, 51), often beginning with QI-first local evidence (32). Governance was limited in scope and unambiguous in stance: automation may deliver standardized information, but clinical decisions remain with the provider, and crisis pathways, disclosure rules, and clarity of liability are preconditions for deployment. This matches the broader digital mental health implementation literature, in which sustainable deployment depends on workflow integration, guidance models, and organizational infrastructure at least as much as efficacy (69–71), and in which human guidance measurably shapes engagement and outcomes of digital interventions (72–75). An embodied telehealth room does not remove the clinician from the loop; on our participants’ account, its value is that it keeps the clinician in the loop with more signal, including for warm handoffs where live introduction measurably improves engagement (20, 53–55). The three postsurvey detractors reinforce the workforce-fit gate from the negative side, with feedback on structural mismatches between the platform and their current practice, evidence that adoption will be role-specific and champion-led rather than uniform.

### 5.5 Design principles revisited and extended for mental health and whole health

Table 11 maps the six design principles from our physical rehabilitation codesign (27) to evidence from this cohort and consolidates two additional principles that emerged in the mental health and whole health context. The prior principles held, but several transformed in emphasis. Embodied guidance extended from movement correction to emotional presence and the non-verbal channel. Minimalist defaults were remotivated by workforce cognitive load, with each removed step treated as a clinical gain. Adaptive accessibility extended beyond input modalities to include bodies and postures, with hands-free and gaze alternatives requested on behalf of veterans with limited fine motor control. Clinical authenticity broadened the most: where rehabilitation stakeholders wanted more measurement, this cohort voted to remove on-screen range-of-motion numbers for mental health sessions while elevating identity-authentic representation (veteran identity markers, older and disabled body types, and personally meaningful environments) to near the top of endorsement, suggesting that authenticity in mental health means the avatar, the environment, and the measurement all match the clinical moment, with representation shipped alongside its safeguards (provider-controlled environment libraries, conservative defaults, and explicit treatment-frame cues). Trust recentered from biomechanical precision to governance precision, and contextual onboarding hardened into a deployment gate with a named time budget. The two new principles were present only faintly in the rehabilitation workshops. They moved to the core of this cohort’s stance: infrastructure-anchored equitable deployment, and information-only automation with the clinician in the loop, the single most consistent position in the data.

### 5.6 Comparison across the codesign series

Across the three workshops, the top priority tracks the population served. Private practice stakeholders (N=24 clinicians, patients, designers, and engineers; Mountain View, CA) voted a minimalist interface and navigation as their top priority (19 votes), followed by expanded assessment and metrics (17 votes), and produced the six design principles revisited in Table 11 (27). Houston VA rehabilitation providers (N=8; four providers paired with four design researchers) prioritized accessibility and adaptation (13 votes), establishing accessibility as a precondition of care and motivating context-aware configuration across delivery models, with this emphasis shaped by the Houston VA rehabilitation context (28). This CRH mental health cohort (N=30; 21 VA CRH stakeholders) recentered minimalist defaults, onboarding, and out-of-the-box readiness (57 votes on 18 ideas), now motivated by workforce cognitive load and home deployment rather than patient novice-ness alone, and elevated identity representation and embodiment (46 votes) to near the top of endorsement, alongside governance limits on automation and infrastructure-anchored deployment as new requirements specific to this cohort. Convergent across all three are onboarding, guidance, and clinical assessment value; divergent is what each context treats as foundational, reflecting each team’s own limits and priorities. This supports context-aware configuration over one-size-fits-all design (28) and suggests that, specifically for mental health, embodiment and identity shift from enhancement to a stakeholder-prioritized differentiator.

### 5.7 Limitations and future work

As with any study, we must consider several limitations that bound these findings. The workshop was convened by the platform’s developer and facilitated in part by platform-affiliated table coaches; we reduced bias through embedded independent SMEs, the exclusion of the product team from usability ratings and gallery voting, anonymous surveys, and the inclusion of negative cases in the analysis. Thematic coding is inherently interpretive and was performed by a single coder with coauthor peer review, but was informed by themes established by the workshop participants. This was also a single-site convening in one CRH region under QI designation, though attending stakeholders were drawn from Boise (VISN 20) and additional regional hubs with national leadership representation; all results remain formative design insights for this cohort. Participants were providers, staff, and subject matter experts rather than direct patients, so veteran-preference findings (identity markers, environments, and connection) are provider-mediated and should be validated with veterans directly; the provider perspective is a reasonable starting point for a platform that must clear workforce and deployment gates first, and veteran-inclusive codesign is the highest-priority extension for addressing this cross-team limit.

Finally, clinician and clinical leadership time is a persistent constraint on this kind of engaged codesign work. Multiday, in-person participation is difficult to sustain against clinical duty, and even among our engaged participants, attendance and attention were interrupted intermittently as time-sensitive clinical issues arose throughout the 3-day workshop. We are grateful for the time and input these participants were able to give. We recognize that this constraint likely shaped both who could fully participate and the depth of engagement possible across all 3 days. This suggests that flexibility in workshop structure, format, and scheduling is not incidental but essential to making this kind of codesign work feasible for busy clinical teams, and should be built into the design of future engagements rather than treated as a workaround when cross-team limits arise.

Future work will include usability testing of new prototypes built from the top-endorsed requirements identified here (minimalist defaults, step-by-step onboarding, identity representation, and environment personalization) with a smaller, more experienced VA cohort, closing the codesign loop from priorities to buildable capability. An IRB-approved VA usability evaluation with veteran participants is already underway for physical therapy, with an operational pathway to extend to whole health. Planned directions also include veteran-inclusive codesign with a standing advisory pathway, framework overlays mapping these findings onto non-adoption and digital health equity determinants (6, 26), and, with partners, a QI-first home-deployment pilot along the collaboration routes participants mapped, with equity-centered metrics for rural reach and device access (56).

## 6 Conclusion

Codesigning embodied XR telehealth with a national VA telehealth program produced a consistent message: when prompted to imagine new capability, stakeholders endorsed minimalist defaults; onboarding; and fit with the infrastructure, workforce, and governance realities that already shape veteran mental healthcare, while affirming embodiment, identity, and personalized environments as the platform’s distinct clinical argument, inseparable from their paired hazards. These findings extend the design principles of our physical rehabilitation codesigns into mental health and whole health, adding identity representation with safeguards and infrastructure-anchored equitable deployment as core requirements for integrated care networks, while recognizing that each team will apply them within its own constraints. Equity-oriented design considerations ran through every day of evidence, from the rural homes the network serves to the people with disabilities and older bodies its avatars must represent, and to the connectivity its sessions must survive. For digital health to be for all, immersive telehealth must be designed from these constraints up, and the stakeholders who deliver that care have shown, in their words, ideas, votes, and priorities, where they would start.

## Data Availability

Anonymized workshop data may be available from the authors upon reasonable request, subject to approval from Immergo Labs and applicable VA data governance, including the Data Use Agreement governing this quality improvement activity. Any shared data will exclude potentially identifiable information. Requests to access the datasets should be directed to AE (aviv@immergolabs.com) or HT (Hilary.Touchett@va.gov).

## Footnotes

^1^We follow VA Immersive in using XR as the umbrella term and reserve VR for fully immersive headset-based experiences. The platform examined in this article supports both fully virtual environments and a passthrough mixed-reality mode, as well as cross-platform access through a web application.

^2^More information at https://www.immergolabs.com.

## Appendix

Workshop materials used in this study (the usability task worksheet, four-axis framework worksheet, importance prioritization worksheet, preworkshop survey, and post-workshop survey) are available at: www.immergolabs.com/boise-vamc-xr-codesign-supl-2026.
